# Smoking and drinking cessation and the risk of oesophageal cancer

**DOI:** 10.1054/bjoc.2000.1274

**Published:** 2000-08-16

**Authors:** C Bosetti, S Franceschi, F Levi, E Negri, R Talamini, C La Vecchia

**Affiliations:** Istituto di Ricerche Farmacologiche ‘Mario Negri’, Via Eritrea 62, Milan, 20157, Italy; Centro di Riferimento Oncologico, Via Pedemontana Occ.le, Aviano (Pordenone), 33081, Italy; Registre Vaudois des Tumeurs, Institut Universitaire de Médecine Sociale et Préventive, CHUV-Falaises 1, Lausanne, 1011, Switzerland; Istituto di Statistica Medica e Biometria, Università degli Studi di Milano, Via Venezian 1, Milan, 20133, Italy

**Keywords:** oesophageal cancer, alcohol drinking, smoking cessation, risk factors, case–control studies

## Abstract

In a case–control study from Italy and Switzerland with 404 oesophageal cancer cases and 1070 hospital controls, the risk of oesophageal cancer declined with time since cessation of smoking or drinking, and was significantly reduced (odds ratio = 0.11) 10 or more years after cessation of both habits. © 2000 Cancer Research Campaign


British Journal of Cancer (2000) **83**(5), 689–691
© 2000 Cancer Research Campaign
doi: 10.1054/ bjoc.2000.1274, available online at http://www.idealibrary.com on IDEAL®

### Smoking and drinking cessation and the risk of oesophageal cancer

**C Bosetti[1], S Franceschi[2], F Levi[3], E Negri[1], R Talamini[2] and C La Vecchia[1,4]**

[1]Istituto di Ricerche Farmacologiche 'Mario Negri', Via Eritrea 62, 20157 Milan, Italy; [2]Centro di Riferimento Oncologico, Via Pedemontana Occ.le, 33081 Aviano (Pordenone), Italy; [3]Registre Vaudois des Tumeurs, Institut Universitaire de Médecine Sociale et Préventive, CHUV-Falaises 1, 1011 Lausanne, Switzerland; [4]Istituto di Statistica Medica e Biometria, Università degli Studi di Milano, Via Venezian 1, 20133 Milan, Italy

**Summary** In a case–control study from Italy and Switzerland with 404 oesophageal cancer cases and 1070 hospital controls, the risk of oesophageal cancer declined with time since cessation of smoking or drinking, and was significantly reduced (odds ratio = 0.11) 10 or more years after cessation of both habits. © 2000 Cancer Research Campaign

**Keywords**: oesophageal cancer; alcohol drinking; smoking cessation; risk factors; case–control studies

While the risk of oesophageal cancer is lower in ex-smokers than in current smokers, and declines steeply with time since stopping smoking (Brown et al, 1988; Yu et al, 1988, La Vecchia et al, 1990; Castellsagué et al, 1999), little is known on the time–risk relation of stopping alcohol drinking on oesophageal carcinogenesis.

In a study conducted in Hong Kong (Cheng et al, 1995) the relative risk (RR) of former drinkers who had stopped for less than 5 years was higher than that of current drinkers, and was significantly below that of current drinkers only 15 or more years after stopping. In a pooled analysis from South America of five studies conducted in high-risk areas (Castellsagué et al, 1999), the RR declined steadily with stopping smoking and drinking, but the decrease in risk was significant only 10 years or more after stopping drinking. In a French study of oesophageal cancer (Launoy et al, 1997) the RR declined 5 or more years after stopping smoking, but no clear pattern of risk was observed after stopping drinking. A similar pattern of risk, with some decline in risk for heavy drinkers only 10 or more years after cessation, and a steeper fall after stopping smoking, was observed in a study of oral and pharyngeal cancer conducted in the same areas of the present investigation (La Vecchia et al, 1999; Franceschi et al, 2000).

To evaluate the pattern of risk after stopping smoking and drinking on oesophageal carcinogenesis, we analysed data from a multicentre case-control study conducted in Italy and Switzerland.

### SUBJECTS AND METHODS

Data of the present analysis derive from two case–control studies of oesophageal cancer conducted between 1992 and 1999 in northern Italy and in the Swiss Canton of Vaud, whose general design has already been described (Bosetti et al, 2000; Levi et al, 2000).

Briefly, cases were subjects admitted to the major teaching and general hospitals in the areas under study with newly-diagnosed, histologically confirmed squamous cell cancer of the oesophagus. A total of 404 subjects (356 men and 48 women) were included, whose median age was 60 years (range, 34–77).

Controls were subjects admitted to the same hospitals as the cases for a wide spectrum of acute, non-neoplastic conditions excluding those related to smoking or alcohol consumption. The control group comprised a total of 1070 subjects, 878 men and 192 women, whose median age was 60 years (range 32–77 years). Twenty-four percent of the controls were admitted for traumas, 27% for other non-traumatic orthopaedic conditions, 31% for acute surgical conditions, and 18% for miscellaneous other illnesses, including eye, nose, ear, skin or dental disorders.

In both studies a structured questionnaire was administered by trained interviewers to cases and controls during their hospital stay, including information on tobacco smoking and alcohol drinking. The section on smoking included questions on smoking status (never, current or ex-smokers), daily number of cigarettes/cigars smoked, age at starting, duration of the habit and, for ex-smokers, age at smoking cessation. Information on alcohol consumption included the average number of days per week each type of alcoholic beverage (wine, beer and spirits) was consumed, and the average number of drinks per day, and, for ex-drinkers, age at drinking cessation. Ex-drinkers and ex-smokers were subjects who had abstained from any type of drinking or smoking for at least 12 months.

Odds ratios (OR) and the corresponding 95% confidence intervals (CI), were estimated using unconditional multiple logistic regression models (Breslow and Day, 1980), including terms for age, sex, study centre, years of education, and amount of alcohol and tobacco consumption.

### RESULTS

Table 1 gives the distribution of the 404 squamous cell oesophageal cancer cases and the 1070 control subjects, according to smoking and drinking status, and time since cessation of these habits. A total of 124 cases were ex-smokers, and 49 were ex-drinkers. Compared to current smokers, the OR for all ex-smokers combined was 0.46 (0.34–0.67). The ORs for ex-smokers were

*Received 6 March 2000*
*Revised 3 April 2000*
*Accepted 3 April 2000*

*Correspondence to:* C Bosetti

**Table 1** Odds ratios[a] (OR) and corresponding 95% confidence interval (CI) of oesophageal cancer according to smoking and drinking status and time since cessation, among 404 cases and 1070 controls, Italy and Switzerland, 1992–1999

| | Cases | Controls | OR (95% CI) |
|---|---|---|---|
| **Tobacco smoking** | | | |
| Current smokers | 238 | 320 | 1[c] |
| Ex-smokers | | | |
| Time since cessation (years) | | | |
| 1–2 | 16 | 19 | 1.37 (0.64–2.96) |
| 3–5 | 27 | 36 | 1.10 (0.60–2.04) |
| 6–9 | 21 | 52 | 0.58 (0.31–1.07) |
| 10–14 | 18 | 85 | 0.31 (0.17–0.56) |
| ≥ 15 | 42 | 182 | 0.31 (0.20–0.49) |
| $\chi^2_1$ for trend | | | 48.76 $P < 0.0001$ |
| Never smokers | 42 | 376 | 0.23 (0.15–0.35) |
| **Alcohol drinking**[b] | | | |
| Current drinkers | 347 | 870 | 1[c] |
| Ex-drinkers | | | |
| Time since cessation (years) | | | |
| 1–5 | 20 | 25 | 0.85 (0.41–1.75) |
| 6–14 | 24 | 19 | 1.72 (0.83–3.57) |
| ≥ 15 | 5 | 22 | 0.53 (0.15–1.85) |
| $\chi^2_1$ for trend | | | 0.03 $P = 0.87$ |
| Never drinkers | 7 | 133 | 0.31 (0.14–0.69) |

[a]Estimates from unconditional logistic regression models, including terms for age, sex, study centre, education, alcohol and tobacco consumption; [b]The sum does not add up to the total because of some missing values; [c]Reference category above unity until 5 years since smoking cessation, and declined to 0.58 for 6–9 years, and to 0.31 for 10 years or more after cessation, approaching that of never-smokers (OR = 0.23) The inverse trend in risk with time since smoking cessation was significant ($P <$ 0.0001).

The risk-reduction according to time since alcohol drinking cessation was less consistent. The ORs for ex-drinkers, as compared to current drinkers, were 0.85 for 1–5 years since drinking cessation, 1.72 for 6–14 years, and 0.53 for 15 or more years since stopping. Thus, only after 15 years since drinking cessation did the OR for ex-drinkers approach that of never-drinkers (OR = 0.31).

The combined effect of stopping either tobacco smoking or alcohol drinking or both, in relation to time since cessation of each habit, is shown in Table 2. As compared to subjects currently both smoking and drinking, the risk of oesophageal cancer decreased with time since smoking cessation across strata of time since drinking cessation. In contrast, a reduced risk of oesophageal cancer in relation to time since drinking cessation was observed only 10 or more years after drinking cessation, except for long term ex-smokers. The OR for having stopped drinking for 10 or more years, adjusted for time since smoking cessation, was 0.37 (95% CI 0.14–0.99) and the OR was more strongly and significantly reduced 10 or more years since cessation of both habits (OR = 0.11, 95% CI 0.01–0.90).

### DISCUSSION

The present study adds further evidence for the well-known effect of tobacco smoking cessation on reducing oesophageal cancer risk (Brown et al, 1988; Yu et al, 1988, La Vecchia et al, 1990; Castellsagué et al, 1999), and it also indicates that stopping alcohol drinking leads to a reduced oesophageal cancer risk after 10 or more years, when allowance is made for time since stopping smoking. It also provides convincing evidence that stopping consumption of both alcohol and tobacco leads to a substantial reduction of oesophageal cancer risk. After 10 or more years since stopping both habits the RR is only about one tenth of that of current smokers and drinkers.

The pattern of risk after stopping drinking does not seem to be linear, and, after allowance for time since smoking cessation, the RR for ex-drinkers was similar or above that of current drinkers for at least 10 years since stopping. This may be due to some characteristics of people who have recently stopped drinking. Most former drinkers were, in fact, heavy drinkers and stopping drinking may therefore, have been influenced by health-related conditions. From a biological viewpoint, the persistence of an excess risk several years after stopping drinking indicates that alcohol is probably not a late-stage carcinogen (Day and Brown, 1980) in this disease, as previously observed for oral and pharyngeal cancers (Franceschi et al, 2000).

In this study information on smoking and alcohol was satisfactorily reproducible (D'Avanzo et al, 1996) and valid (Ferraroni et al, 1996), but no information was available on reliability of data on time since stopping habits. The hospital setting is likely, however, to ensure a reasonable comparability of information collected (Breslow and Day, 1980). With reference to confounding, careful allowance was made for tobacco and alcohol, and their related time factors.

In these southern European populations about 60% of oesophageal cancer cases could be attributed, after reciprocal allowance in multivariate analysis, to tobacco smoking, 80% to alcohol drinking, and 88% to the combination of the two habits

**Table 2** Combined effect of time since smoking and drinking cessation on oesophageal cancer,[a] Italy and Switzerland, 1992–1999

| Time since drinking cessation (years) | Time since smoking cessation (years): Current | 1–9 | ≥ 10 | OR[c] for drinking cessation |
|---|---|---|---|---|
| Current | 1[b] | 0.71 (0.46–1.09) | 0.28 (0.19–0.42) | 1[b] |
| 1–9 | 2.09 (0.79–5.53) | 1.11 (0.37–3.30) | 0.04 (0.01–0.33) | 1.28 (0.67–2.43) |
| ≥ 10 | 0.30 (0.08–1.11) | 0.44 (0.06–3.13) | 0.11 (0.01–0.90) | 0.37 (0.14–0.99) |
| OR[d] for smoking cessation | 1[b] | 0.72 (0.48–1.08) | 0.26 (0.18–0.38) | |

[a]Estimates from unconditional logistic regression models, including terms for age, sex, study centre, education, alcohol and tobacco consumption; [b]Reference category; [c]Adjusted also for time since smoking cessation; [d]Adjusted also for time since drinking cessation

 © 2000 Cancer Research Campaign

(Bruzzi et al, 1985). Stopping alcohol drinking, and particularly cessation of smoking, could therefore have an appreciable impact in reducing oesophageal cancer risk, and therefore represent obvious priorities for prevention and public-health purposes.

### ACKNOWLEDGEMENTS

This work was conducted with the contribution of the Italian Association for Cancer Research, Milan, Italy and the Swiss Foundation for Research Against Cancer. The authors thank Mrs M Paola Bonifacino for editorial assistance.

### REFERENCES

Bosetti C, La Vecchia C, Talamini R, Simonato L, Zambon P, Negri E, Trichopoulos D, Lagiou P, Bardini R and Franceschi S (2000) Food groups and risk of squamous cell esophageal cancer in northern Italy. *Int J Cancer*, in press

Breslow NE and Day NE (1980) *Statistical methods in cancer research*, Vol. 1. *The analysis of case-control studies*, pp. 32. IARC Scientific Publications: Lyon, France

Brown LM, Blot WJ, Schuman SH, Smith VM, Ershow AG, Marks RD and Fraumeni JF Jr (1988) Environmental factors and high risk of oesophageal cancer among men in coastal South Carolina. *J Natl Cancer Inst* **80**: 1620–1625

Bruzzi P, Green SB, Byar DP, Brinton LA and Schairer C (1985) Estimating the population attributable risk for multiple risk factors using case-control data. *Am J Epidemiol* **122**: 904–914

Castellsagué X, Muñoz N, De Stefani E, Victora CG, Castelletto R, Rolón PA, and Quintana MJ (1999) Independent and joint effects of tobacco smoking and alcohol drinking on the risk of esophageal cancer in men and women. *Int J Cancer* **82**: 657–664

Cheng KK, Duffy SW, Day NE, Lam TH, Chung SF and Badrinath P (1995) Stopping drinking and risk of oesophageal cancer. *BMJ* **310**: 1094–1097

D'Avanzo B, La Vecchia C, Katsouyanni K, Negri E and Trichopoulos D (1996) Reliability of information on cigarette smoking and beverage consumption provided by hospital controls. *Epidemiology* **5**: 312–315

Day ND and Brown CC (1980) Multistage models and primary prevention of cancer. *J Natl Cancer Inst* **64**: 977–989

Ferraroni M, Decarli A, Franceschi S, La Vecchia C, Enard L, Negri E, Parpinel M and Salvini S (1996) Validity and reproducibility of alcohol consumption in Italy. *Int J Epidemiol* **25**: 775–782

Franceschi S, Levi F, Dal Maso L, Talamini R, Conti E, Negri E and La Vecchia C (2000) Cessation of alcohol drinking and risk of cancer of the oral cavity and pharynx. *Int J Cancer* **85**: 787–790

Launoy G, Milan CH, Faivre J, Pienkowski P, Milan CI and Gignoux M (1997) Alcohol, tobacco and oesophageal cancer: effects of the duration of consumption, mean intake and current and former consumption. *Br J Cancer* **75**: 1389–1396

La Vecchia C, Bidoli E, Barra S, D'Avanzo B, Negri E, Talamini R and Franceschi S (1990) Type of cigarettes and cancers of the upper digestive and respiratory tract. *Cancer Causes Control* **1**: 69–74

La Vecchia C, Franceschi S, Bosetti C, Levi F, Talamini R and Negri E (1999) Time since stopping smoking and risk of oral and pharyngeal cancers *J Natl Cancer Inst* **91**: 726–728

Levi F, Pasche F, Lucchini F, Bosetti C, Franceschi S, Monnier P and La Vecchia C (2000) Food groups and oesophageal cancer risk in Vaud, Switzerland. *Eur J Cancer Prev*, in press

Yu MC, Garabrant DH, Peters JM and Mack TM (1988) Tobacco, alcohol, diet, occupation, and carcinoma of the esophagus. *Cancer Res* **48**: 3843–3848

*© 2000 Cancer Research Campaign*
